# Supplementary material for: Metalation calculators for E. coli strain JM109 (DE3): aerobic, anaerobic, and hydrogen peroxide exposed cells cultured in LB media
Source: Metallomics. 2022 Aug 6;14(9):mfac058. doi: 10.1093/mtomcs/mfac058 (PMC9434800; doi:10.1093/mtomcs/mfac058)
Supplement: mfac058_Supplemental_Files [file mfac058_supplemental_files.zip › Metallation Calculators SUPPLEMENTARY DATA.docx]

Supplementary Data to Metalation Calculators

**Metalation calculators for *E. coli* strain JM109 (DE3): Aerobic, anaerobic and hydrogen peroxide exposed cells cultured in LB media**

Andrew W. Foster^1,2^, Sophie E. Clough^1,2^, Tessa R. Young^1,2^, Alison R. Clarke^3^, Nigel J. Robinson^1,2*^

^1^ Department of Biosciences, Durham University, Durham, UK

^2^ Department of Chemistry, Durham University, Durham, UK

^3^ Advanced Research Computing, Durham University, Durham, UK

*[nigel.robinson@durham.ac.uk](mailto:nigel.robinson@durham.ac.uk)

**Supplementary Table 1**. qPCR primers used in this work.

| No. | Name | Sequence | Product size (bp) | Reference |
| --- | --- | --- | --- | --- |
| 1 | *mntS*_F | 5’-GTATGCGCGTGTTTAGTCATTC-3’ | 105 | This work |
| 2 | *mntS*_R | 5’-TATCGGAAGGTTTATCTTGCTG-3’ | 105 | This work |
| 3 | *fepD*_F | 5’-TGCAAACCCTCACCCGAAAC-3’ | 111 | This work |
| 4 | *fepD*_R | 5’-GCGCGGAAGAGTAACCAAACAG-3’ | 111 | This work |
| 5 | *rcnA*_F | 5’-GAACCAGGGCACTCAAAAAC-3’ | 108 | Ref [1] |
| 6 | *rcnA*_R | 5’-TGCGGTATGCGAAATAGTTG-3’ | 108 | Ref [1] |
| 7 | *nikA*_F | 5’-AACCCGCACCTTTACACGCC-3’ | 114 | This work |
| 8 | *nikA*_R | 5’-AGTCCAGCTTTTTGCCAGCC-3’ | 114 | This work |
| 9 | *znuA*_F | 5’-GTTTGGACTGACACCGCTTG-3’ | 111 | Ref [2] |
| 10 | *znuA*_R | 5’-ACGCAGGTTGCTTTTTGCTC-3’ | 111 | Ref [2] |
| 11 | *zntA*_F | 5’-CGAAGCACAGGTTGCTGAAC-3’ | 114 | This work |
| 12 | *zntA*_R | 5’-CCGGCAGCGCAAATCAATAC-3’ | 114 | This work |
| 13 | *copA*_F | 5’-GTCACAAACTATCGACCTGACCC-3’ | 104 | This work |
| 14 | *copA*_R | 5’-CATCCGCCTGCTCAACATCC-3’ | 104 | This work |
| 15 | *rpoD*_F | 5’-GTGGCTTGCAGTTCCTTGAC-3’ | 108 | Ref [2] |
| 16 | *rpoD*_R | 5’-AGGTTGCGTAGGTGGAGAAC-3’ | 108 | Ref [2] |
| 17 | *gyrA*_F | 5’-AGGGCTGATGGAACACATCC-3’ | 108 | This work |
| 18 | *gyrA*_R | 5’-ATATACACCTTGCCGCGACC-3’ | 108 | This work |

**Supplementary Table 2**. Rationale for choice of growth conditions for determining sensor calibration boundary conditions.

| +/- O_2_ | Treatment | Exposure time (min) | Concentration (mM) | Rationale |
| --- | --- | --- | --- | --- |
| + | Mn | 120 | 0.5, 2, 4 | No growth inhibition observed upon Mn treatment, indeed higher concentrations of Mn appeared to enhance growth. A range of Mn concentrations from the growth experiment were sampled. |
| + | H_2_O_2_ | 60, 120 | 0.1 | *mntH* (Mn import) expression is upregulated by OxyR in response to H_2_O_2_ treatment. Maximal induction observed following 45-60 min treatment with 100 µM H_2_O_2_ in *Salmonella* and similar results observed in *E. coli* [3,4]. Hypothesised that this treatment could increase intracellular Mn availability more than exposure to Mn alone. |
| + | H_2_O_2_ / Mn | 60, 120 | 0.1 / 4 | Hypothesised that H_2_O_2_ exposure in the presence of Mn might give maximum intracellular Mn and therefore lowest expression of *mntS*. |
| + | EDTA | 120 | 0.5 | 16% growth inhibition relative to untreated cultures. |
| + | EDTA | 20, 60, 120, 240 | 1 | To minimise metal availability in growth medium increased [EDTA] to 1 mM and varied exposure times. 1 mM EDTA previously used for calibration of Co and Zn availability in *E. coli* producing B_12_ [2]. |
| + | Fe | 120 | 0.5 | 19% growth inhibition relative to untreated cells. |
| + | Fe | 120 | 1.5 | Increased [Fe] to maximise intracellular Fe availability. |
| + | Fe | 120 | 4 | Increased [Fe] to maximise intracellular Fe availability. |
| + | Co | 120 | 0.5 | 17% growth inhibition relative to untreated cells. Obtained a similar maximum fold induction of *rcnA* to that observed previously [2]. |
| + | Co | 10 | 0.5 | Hypothesised that maximum response of *rcnA* might be observed in response to ‘cobalt shock’. |
| + | Ni | 120 | 0.5 | Here a growth condition was selected which gave negligible growth inhibition relative to untreated cells (3%) due to an initial misinterpretation of growth data. However, comparison of *rpoD* *C*q for Ni exposed cultures with the control condition suggest that this is the maximum permissible [Ni] for this analysis (Supplementary Table 3). |
| + | Ni | 120 | 2 | Increased [Ni] to maximise intracellular Ni availability. |
| + | Ni | 120 | 3 | Increased [Ni] to maximise intracellular Ni availability. |
| + | Zn | 120 | 0.6 | 21% growth inhibition relative to untreated cultures. |
| + | Zn | 60, 120 | 1 | Increased [Zn] to maximise intracellular Zn availability. |
| + | Zn | 120 | 0.8 | ~50% growth inhibition relative to untreated cultures. Included as 60 and 120 min treatment with 1 mM Zn resulted in aberrant expression of *rpoD*. |
| + | Zn | 10 | 1, 2 | Hypothesised that maximum response of *zntA* might be observed in response to ‘zinc shock’. |
| + | Cu | 120 | 1.8 | 14% growth inhibition relative to untreated cultures. |
| + | Cu | 120 | 2.4 | Increased [Cu] to maximise intracellular Cu availability. 36% growth inhibition relative to untreated cultures. Aberrant expression of *rpoD* had been observed in response to [Ni] and [Zn] which resulted in >50% growth inhibition relative to untreated cultures so a less inhibitory concentration was chosen here. |
| + | Cu | 10 | 2.4 | Hypothesised that maximum response of *copA* might be observed in response to ‘copper shock’. |
| - | Fe | 120, 180 | 0.5 | Fe availability may be higher under anaerobic conditions [5,6]. Anaerobic sachets used reduce O_2_ to <1 % within 30 min and <0.1% within 3 h. [Fe] selected based on earlier results. |
| - | Ni | 120 | 0.5 | [Ni] chosen by analogy to aerobic experiments. |
| - | Ni | 120 | 1.5, 2 | Increased [Ni] to maximise intracellular Ni availability with <50% growth inhibition. |
| - | EDTA | 120 | 1 | [EDTA] chosen by analogy to aerobic experiments. |
| - | DMG | 120 | 0.5, 1 | DMG used as Ni specific chelator for maximum expression of *nikA* under anaerobic conditions. 0.5 and 1 mM DMG treatment results in a significant reduction in hydrogenase activity in anaerobic *Salmonella* [7]. |

**Supplementary Table 3**. *rpoD* *C*_q_ difference values*^a^*.

| +/- O_2_ | Treatment | Exposure time (min) | *rpoD* *C*_q_ difference relative to untreated aerobic samples | *rpoD* *C*_q_ difference relative to corresponding (120 or 180 min) anaerobic untreated samples |
| --- | --- | --- | --- | --- |
| +O_2_ | 0.5 mM EDTA | 120 | 0.33(±0.5) | n.a. |
| +O_2_ | 1 mM EDTA | 20 | 2.0(±0.1) | n.a. |
| +O_2_ | 1 mM EDTA | 60 | -0.56(±0.2) | n.a. |
| +O_2_ | 1 mM EDTA | 120 | 0.040(±0.2) | n.a. |
| +O_2_ | 1 mM EDTA | 240 | 0.15(±0.1) | n.a. |
| +O_2_ | 0.5 mM Mn | 120 | 1.3(±0.3) | n.a. |
| +O_2_ | 2 mM Mn | 120 | 1.7(±0.3) | n.a. |
| +O_2_ | 4 mM Mn | 120 | 1.2(±0.09) | n.a. |
| +O_2_ | 100 µM H_2_O_2_ | 60 | 0.86(0.3) | n.a. |
| +O_2_ | 100 µM H_2_O_2_ | 120 | 1.5(±0.3) | n.a. |
| +O_2_ | 100 µM H_2_O_2_  4 mM Mn | 60 | 1.2(±0.6) | n.a. |
| +O_2_ | 100 µM H_2_O_2_  4 mM Mn | 120 | 0.35(±1) | n.a. |
| +O_2_ | 0.5 mM Fe | 120 | 0.82(±0.1) | n.a. |
| +O_2_ | 0.5 mM Co | 120 | -0.57(±0.1) | n.a. |
| +O_2_ | 0.5 mM Co | 10 | 3.4(±0.5) | n.a. |
| +O_2_ | 0.5 mM Ni | 120 | 1.9(±0.6) | n.a. |
| +O_2_ | 2 mM Ni | 120 | 2.0(±0.1) | n.a. |
| +O_2_ | 3 mM Ni | 120 | 3.2(±0.2) | n.a. |
| +O_2_ | 0.6 mM Zn | 120 | 0.50(±0.08) | n.a. |
| +O_2_ | 0.8 mM Zn | 120 | 0.89(±0.4) | n.a. |
| +O_2_ | 1 mM Zn | 10 | 2.8(±0.2) | n.a. |
| +O_2_ | 1 mM Zn | 60 | 2.7(±0.1) | n.a. |
| +O_2_ | 1 mM Zn | 120 | 3.2(±0.4) | n.a. |
| +O_2_ | 2 mM Zn | 10 | 1.3(±0.1) | n.a. |
| +O_2_ | 1.8 mM Cu | 120 | 1.3(±0.1) | n.a. |
| +O_2_ | 2.4 mM Cu | 120 | 0.97(±0.2) | n.a. |
| +O_2_ | 2.4 mM Cu | 10 | 2.2(±0.8) | n.a. |
| -O_2_ | 0.5 mM Fe | 120 | -0.37(±0.2) | 0.43(±0.2) |
| -O_2_ | 0.5 mM Fe | 180 | 2.8(±0.3) | 0.17(±0.3) |
| -O_2_ | 0.5 mM DMG | 120 | 1.3(±0.04) | 1.8(±0.05) |
| -O_2_ | 1 mM DMG | 120 | 1.4(±0.1) | 1.8(±0.1) |
| -O_2_ | 1 mM EDTA | 120 | -0.98(±0.4) | -0.50(±0.4) |
| -O_2_ | Untreated | 120 | -0.82(±0.06) | n.a. |
| -O_2_ | Untreated | 180 | 2.7(±0.2) | n.a. |
| -O_2_ | 0.5 mM Ni | 120 | 0.53(±0.8) | 0.98(±0.8) |
| -O_2_ | 1.5 mM Ni | 120 | -0.50(±0.1) | -0.026(±0.1) |
| -O_2_ | 2 mM Ni | 120 | 0.49(±0.2) | 0.96(±0.2) |

^a^ *rpoD* *C*_q_ difference between values determined for treated samples (and anaerobic untreated samples) and the mean value determined for untreated aerobic cultures and, where appropriate, relative to the mean value determined for the corresponding untreated anaerobic cultures. Values shown as mean ± standard deviation.

**Supplementary Table 4**. *gyrA* *C*_q_ difference values*^a^*.

| +/- O_2_ | Treatment | Exposure time (min) | *gyrA* *C*_q_ difference relative to untreated aerobic samples | *gyrA* *C*_q_ difference relative to corresponding (180 min) anaerobic untreated samples |
| --- | --- | --- | --- | --- |
| +O_2_ | 1 mM Zn | 120 | 1.5(±0.7) | n.a. |
| -O_2_ | Untreated | 180 | 0.90(±0.04) | n.a. |
| -O_2_ | 0.5 mM Fe | 180 | 1.1(±0.2) | 0.24(±0.2) |

^a^ *gyrA* *C*_q_ difference between values determined for treated samples (and anaerobic untreated samples) and the mean value determined for untreated aerobic cultures plus, where appropriate, relative to the mean value determined for the corresponding untreated anaerobic cultures. Values shown as mean ± standard deviation.

**Supplementary Table 5**. List of target genes for *E. coli* metal sensors*^a^*.

| Sensor | Targets | Total |
| --- | --- | --- |
| MntR | *mntS*/*R*  *mntH*  *mntP*  *dps* | 4 |
| Fur | *fepD* | 37 |
| RcnR | *rcnA* | 1 |
| NikR | *nikA* | 1 |
| CueR | *copA*  *cueO* | 2 |
| ZntR | *zntA* | 1 |
| Zur | *znuABC*  *zinT*  *L31P*  *pliG* | 4 |

*^a^* Experimentally validated targets for each metal sensor in *E. coli* [8-14]. For Fur the size of the regulon was estimated from the number of transcriptional units with regulatory sequences bound by Fur during exposure to iron-replete, but not iron-starvation conditions [15,16]. *fepD* is shown as a representative target.

**Supplementary Table 6**. Δ*C*_q_ values obtained from qPCR analysis of indicated samples with primers specific to *nikA* and *rpoD* (control)^a^.

| +/- O_2_ | Treatment | Exposure time (min) | Δ*C*_q_ *_nikA_*_-_*_rpoD_* | *rpoD* *C*_q_ difference relative to untreated aerobic samples <2? Yes/No | *rpoD* *C*_q_ difference relative to corresponding (120 min) anaerobic untreated samples <2? Yes/No |
| --- | --- | --- | --- | --- | --- |
| -O_2_ | 1 mM DMG | 120 | 0.27(±0.2) | Y | Y |
| -O_2_ | 0.5 mM DMG | 120 | 0.38(±0.3) | Y | Y |
| -O_2_ | 1 mM EDTA | 120 | 0.48(±0.2) | Y | Y |
| -O_2_ | Untreated | 120 | 0.78(±0.1) | Y | n.a |
| +O_2_ | 1 mM EDTA | 60 | 1.6(±0.5) | Y | n.a. |
| +O_2_ | 1 mM EDTA | 120 | 2.1(±0.4) | Y | n.a. |
| +O_2_ | Untreated | 120 | 2.3(±0.4) | n.a. | n.a. |
| +O_2_ | 1 mM EDTA | 240 | 2.7(±0.5) | Y | n.a. |
| +O_2_ | 0.5 mM EDTA | 120 | 3.4(±0.5) | Y | n.a. |
| +O_2_ | 100 µM H_2_O_2_ | 120 | 3.6(±0.3) | Y | n.a. |
| -O_2_ | 1.5 mM Ni | 120 | 4.5(±0.1) | Y | Y |
| -O_2_ | 0.5 mM Ni | 120 | 5.1(±0.4) | Y | Y |
| -O_2_ | 2 mM Ni | 120 | 5.9(±0.3) | Y | Y |
| +O_2_ | 2 mM Ni | 120 | 6.7(±0.3) | N | n.a. |
| +O_2_ | 3 mM Ni | 120 | 7.1(±0.3) | N | n.a. |
| +O_2_ | 0.5 mM Ni | 120 | 7.2(±0.2) | Y | n.a. |

^a^ Δ*C*_q_ *_nikA_*_-_*_rpoD_* obtained for each biological replicate, mean (± standard deviation). Data from samples with a difference in *rpoD* *C*_q_ greater than 2 relative to untreated aerobic samples were not processed further.

**Supplementary Table 7**. Growth of *E. coli* strain JM109 (DE3) in response to various treatments*^a^*.

|  |  |  | % growth relative to untreated | | | | |
| --- | --- | --- | --- | --- | --- | --- | --- |
| +/- O_2_ | Treatment | Concentration (mM) | 20 min | 60 min | 120 min | 180 min | 240 min |
| +O_2_ | MnCl_2_ | 0.5 | - | 97(±2) | 105(±11) | 101(±2) | 101(±6) |
| +O_2_ | MnCl_2_ | 1 | - | 151(±1) | 155(±5) | 154(±4) | 136(±5) |
| +O_2_ | MnCl_2_ | 1.5 | - | 149(±5) | 144(±5) | 145(±7) | 127(±3) |
| +O_2_ | MnCl_2_ | 2 | - | 111(±12) | 101(±7) | 108(±7) | 103(±3) |
| +O_2_ | MnCl_2_ | 3 | - | 238(±3) | 214(±13) | 191(±7) | 161(±12) |
| +O_2_ | MnCl_2_ | 4 | - | 232(±6) | 184(±2) | 171(±11) | 142(±2) |
| +O_2_ | FeSO_4_ | 0.5 | - | 91(±1) | 81(±1) | 83(±0) | 89(±0) |
| +O_2_ | FeSO_4_ | 1 | - | 69(±2) | 64(±0) | 64(±1) | 67(±4) |
| +O_2_ | FeSO_4_ | 1.5 | - | 64(±2) | 59(±3) | 57(±0) | 60(±3) |
| +O_2_ | FeSO_4_ | 2 | - | 95(±2) | 98(±3) | 94(±4) | 92(±5) |
| +O_2_ | FeSO_4_ | 3 | - | 107(±2) | 84(±9) | 67(±6) | 56(±5) |
| +O_2_ | FeSO_4_ | 4 | - | 107(±4) | 85(±2) | 69(±3) | 56(±2) |
| +O_2_ | CoCl_2_ | 0.1 | - | 88(±2) | 101(±1) | 97(±2) | 91(±4) |
| +O_2_ | CoCl_2_ | 0.5 | - | 78(±0) | 83(±1) | 82* | 74(±1) |
| +O_2_ | CoCl_2_ | 1 | - | 57(±2) | 46(±1) | 36(±2) | 32(±1) |
| +O_2_ | NiSO_4_ | 0.5 | - | 93(±0) | 97(±5) | 92(±1) | 105(±3) |
| +O_2_ | NiSO_4_ | 1 | - | 74(±3) | 95(±7) | 75(±9) | 82(±9) |
| +O_2_ | NiSO_4_ | 1.5 | - | 76(±7) | 82(±7) | 68(±2) | 73(±2) |
| +O_2_ | NiSO_4_ | 2 | - | 63(±4) | 41(±5) | 40(±9) | 38(±8) |
| +O_2_ | NiSO_4_ | 2.5 | - | 60(±2) | 34(±8) | 30(±5) | 21(±2) |
| +O_2_ | NiSO_4_ | 3 | - | 50(±15) | 37(±14) | 31(±12) | 22(±0) |
| +O_2_ | CuSO_4_ | 0.1 | - | 104(±5) | 119(±7) | 109(±3) | 108(±1) |
| +O_2_ | CuSO_4_ | 0.5 | - | 103(±2) | 116(±7) | 108(±5) | 106(±2) |
| +O_2_ | CuSO_4_ | 1 | - | 97(±1) | 110(±8) | 97(±3) | 96(±1) |
| +O_2_ | CuSO_4_ | 1.5 | - | 92(±4) | 87(±1) | 94(±5) | 95(±7) |
| +O_2_ | CuSO_4_ | 1.8 | - | 92(±3) | 86(±2) | 94(±7) | 96(±1) |
| +O_2_ | CuSO_4_ | 2 | - | 95(±5) | 90(±1) | 94(±4) | 94(±2) |
| +O_2_ | CuSO_4_ | 2.2 | - | 67(±2) | 64(±1) | 69(±6) | 84(±2) |
| +O_2_ | CuSO_4_ | 2.3 | - | 72(±1) | 66(±1) | 72(±3) | 83(±2) |
| +O_2_ | CuSO_4_ | 2.4 | - | 70(±1) | 64(±1) | 67(±2) | 74(±7) |
| +O_2_ | CuSO_4_ | 2.5 | - | 65(±2) | 67(±2) | 77(±1) | 90(±2) |
| +O_2_ | CuSO_4_ | 3 | - | 61(±2) | 54(±1) | 60(±4) | 67(±4) |
| +O_2_ | CuSO_4_ | 4 | - | 52(±4) | 42(±3) | 40(±1) | 39(±1) |
| +O_2_ | ZnSO_4_ | 0.05 | - | 97(±1) | 99(±2) | 100(±3) | 106(±2) |
| +O_2_ | ZnSO_4_ | 0.1 | - | 103(±2) | 103(±2) | 100(±3) | 105(±2) |
| +O_2_ | ZnSO_4_ | 0.5 | - | 97(±1) | 95(±3) | 96(±3) | 104(±2) |
| +O_2_ | ZnSO_4_ | 0.6 | - | 70(±2) | 79(±1) | 86(±4) | 92(±1) |
| +O_2_ | ZnSO_4_ | 0.7 | - | 55(±4) | 58(±7) | 72(±5) | 79(±1) |
| +O_2_ | ZnSO_4_ | 0.8 | - | 44(±3)^b^ 48(±2)^c^ | 56(±5)^b^  43(±2)^c^ | 71(±1)^b^  57(±5)^c^ | 82(±4)^b^  66(±3)^c^ |
| +O_2_ | ZnSO_4_ | 1 | - | 23(±3) | 20(±2) | 30(±5) | 50(±3) |
| +O_2_ | ZnSO_4_ | 1.5 | - | 44(±5) | 14(±4) | 33(±37) | 15(±8) |
| +O_2_ | EDTA | 0.1 | - | 81(±1) | 92(±3) | 91(±7) | 98(±1) |
| +O_2_ | EDTA | 0.3 | - | 82(±2) | 87(±6) | 92(±3) | 91(±3) |
| +O_2_ | EDTA | 0.5 | - | 88(±1) | 84(±2) | 84(±2) | 88(±1) |
| +O_2_ | EDTA | 0.7 | - | 76(±1) | 69(±3) | 72(±2) | 73(±1) |
| +O_2_ | EDTA | 1 | 81(±13) | 90(±7) | 89(±3) | 100(±9) | 79(±3) |
| +O_2_ | EDTA | 1.3 | - | 73(±2) | 68(±3) | 70(±0) | 71(±1) |
| +O_2_ | EDTA | 2 | - | 72(±2) | 68(±0) | 66(±2) | 70(±2) |
| +O_2_ | H_2_O_2_ | 0.1 | - | 85(±5) | 87(±4) | 91(±2) | 90(±1) |
| +O_2_ | H_2_O_2_ / MnCl_2_ | 0.1 / 4 | - | 92(±5) | 94(±8) | 103(±6) | 87(±4) |
| -O_2_ | EDTA | 1 | - | - | 70(±2) | - | - |
| -O_2_ | DMG | 0.5 | - | - | 111(±2) | - | - |
| -O_2_ | DMG | 1 | - | - | 104(±0.5) | - | - |
| -O_2_ | FeSO_4_ | 0.5 | - | - | 113(±4) | 111(±7) | - |
| -O_2_ | NiSO_4_ | 0.5 | - | - | 106(±2) | - | - |
| -O_2_ | NiSO_4_ | 1.5 | - | - | 54(±5) | - | - |
| -O_2_ | NiSO_4_ | 2 | - | - | 61(±14) | - | - |

^a^ Mean growth of n = 3 biological replicates (± standard deviation) relative to mean of untreated cultures (n = 3 biological replicates) (for anaerobic 180 min treatment with 0.5 mM FeSO_4_, n = 5 biological replicates for both treated and untreated cultures).

* One of the biological replicates in this experiment became contaminated, average of two biological replicates presented.

^b, c^ This treatment performed with 3 biological replicates on two separate occasions.

**Supplementary Table 8**. Δ*C*_q_ values obtained from qPCR analysis of indicated samples with primers specific to *mntS* and *rpoD* (control)^a^.

| +/- O_2_ | Treatment | Exposure time (min) | Δ*C*_q_ *_mntS_*_-_*_rpoD_* | *rpoD* *C*_q_ difference relative to untreated aerobic samples <2? Yes/No |
| --- | --- | --- | --- | --- |
| +O_2_ | 1 mM EDTA | 60 | 0.16(±0.5) | Y |
| +O_2_ | 1 mM EDTA | 240 | 0.66(±0.08) | Y |
| +O_2_ | 1 mM EDTA | 120 | 0.82(±0.2) | Y |
| +O_2_ | 0.5 mM EDTA | 120 | 0.92(±0.03) | Y |
| +O_2_ | Untreated | 120 | 1.8(±0.7) | n.a. |
| -O_2_ | Untreated | 120 | 2.3(±0.3) | Y |
| +O_2_ | 1 mM EDTA | 20 | 3.3(±0.2) | N |
| +O_2_ | 0.5 mM Mn | 120 | 3.9(±0.3) | Y |
| +O_2_ | 2 mM Mn | 120 | 4.1(±0.3) | Y |
| +O_2_ | 4 mM Mn | 120 | 4.3(±0.4) | Y |
| +O_2_ | 100 µM H_2_O_2_ | 60 | 5.0(±0.3) | Y |
| +O_2_ | 100 µM H_2_O_2_ | 120 | 5.2(±0.3) | Y |
| +O_2_ | 100 µM H_2_O_2_  4 mM Mn | 120 | 6.4(±0.7) | Y |
| +O_2_ | 100 µM H_2_O_2_  4 mM Mn | 60 | 6.9(±0.3) | Y |

^a^ Δ*C*_q_ *_mntS_*_-_*_rpoD_* obtained for each biological replicate, mean (± standard deviation). Data from samples with a difference in *rpoD* *C*_q_ greater than 2 relative to untreated aerobic samples were not processed further.

**Supplementary Table 9**. Δ*C*_q_ values obtained from qPCR analysis of indicated samples with primers specific to *fepD* and *rpoD* (control)^a^.

| +/- O_2_ | Treatment | Exposure time (min) | Δ*C*_q_ *_fepD_*_-_*_rpoD_* | *rpoD* *C*_q_ difference relative to untreated aerobic samples <2? Yes/No | *rpoD* *C*_q_ difference relative to corresponding (120 or 180 min) anaerobic untreated samples <2? Yes/No |
| --- | --- | --- | --- | --- | --- |
| +O_2_ | 1 mM EDTA | 60 | 1.8(±0.1) | Y | n.a. |
| +O_2_ | 1 mM EDTA | 120 | 2.4(±0.6) | Y | n.a. |
| +O_2_ | 0.5 mM EDTA | 120 | 2.7(±0.3) | Y | n.a. |
| +O_2_ | 1 mM EDTA | 240 | 2.8(±0.1) | Y | n.a. |
| +O_2_ | 1 mM EDTA | 20 | 3.2(±0.3) | N | n.a. |
| -O_2_ | Untreated | 120 | 6.1(±0.4) | Y | n.a. |
| -O_2_ | 0.5 mM Fe | 120 | 7.0(±0.04) | Y | Y |
| +O_2_ | Untreated | 120 | 7.6(±0.16) | n.a. | n.a. |
| +O_2_ | 0.5 mM Fe | 120 | 7.9(±0.1) | Y | n.a. |
| +O_2_ | 100 µM H_2_O_2_ | 120 | 8.5(±0.4) | Y | n.a. |
| -O_2_ | Untreated | 180 | 8.7(±0.3) | N | n.a |
| -O_2_ | 0.5 mM Fe | 180 | 9.1(±0.3) | N | Y |

^a^Δ*C*_q_ *_fepD_*_-_*_rpoD_* obtained for each biological replicate, mean (± standard deviation). Data from samples with a difference in *rpoD* *C*_q_ greater than 2 relative to untreated aerobic samples were not processed further, in samples treated with 0.5 mM FeSO_4_ for 180 min or equivalent untreated samples this difference was not replicated with the use of an alternative control gene, *gyrA* (Supplementary Table 4, Methods).

**Supplementary Table 10**. Δ*C*_q_ values obtained from qPCR analysis of indicated samples with primers specific to *rcnA* and *rpoD* (control)^a^.

| +/- O_2_ | Treatment | Exposure time (min) | Δ*C*_q_ *_rcnA_*_-_*_rpoD_* | *rpoD* *C*_q_ difference relative to untreated aerobic samples <2? Yes/No |
| --- | --- | --- | --- | --- |
| +O_2_ | 0.5 mM Co | 120 | 2.9(±0.4) | Y |
| +O_2_ | 0.5 mM Co | 10 | 4.2(±0.3) | N |
| +O_2_ | 1 mM EDTA | 60 | 6.2(±0.2) | Y |
| +O_2_ | 1 mM EDTA | 120 | 6.5(±0.2) | Y |
| +O_2_ | 1 mM EDTA | 240 | 6.8(±0.3) | Y |
| +O_2_ | 0.5 mM EDTA | 120 | 7.1(±0.2) | Y |
| -O_2_ | Untreated | 120 | 7.9(±0.09) | Y |
| +O_2_ | 1 mM EDTA | 20 | 7.9(±0.3) | N |
| +O_2_ | Untreated | 120 | 8.1(±0.3) | n.a. |
| +O_2_ | 100 µM H_2_O_2_ | 120 | 9.5(±0.5) | Y |

^a^ Δ*C*_q_ *_rcnA_*_-_*_rpoD_* obtained for each biological replicate, mean (± standard deviation). Data from samples with a difference in *rpoD* *C*_q_ greater than 2 relative to untreated aerobic samples were not processed further.

**Supplementary Table 11**. Δ*C*_q_ values obtained from qPCR analysis of indicated samples with primers specific to *znuA* and *rpoD* (control)^a^.

| +/- O_2_ | Treatment | Exposure time (min) | Δ*C*_q_ *_znuA_*_-_*_rpoD_* | *rpoD* *C*_q_ difference relative to untreated aerobic samples <2? Yes/No |
| --- | --- | --- | --- | --- |
| +O_2_ | 1 mM EDTA | 60 | -2.1(±0.7) | Y |
| +O_2_ | 1 mM EDTA | 240 | -2.0(±0.1) | Y |
| +O_2_ | 1 mM EDTA | 120 | -1.9(±0.3) | Y |
| +O_2_ | 1 mM EDTA | 20 | -1.8(±0.1) | N |
| +O_2_ | 0.8 mM Zn | 120 | -1.7(±0.4) | Y |
| +O_2_ | 0.5 mM EDTA | 120 | -1.4(±0.8) | Y |
| +O_2_ | 0.6 mM Zn | 120 | 1.7(±0.1) | Y |
| -O_2_ | Untreated | 120 | 2.1(±0.2) | Y |
| +O_2_ | Untreated | 120 | 2.2(±0.3) | n.a. |
| +O_2_ | 100 µM H_2_O_2_ | 120 | 4.3(±0.6) | Y |
| +O_2_ | 1 mM Zn | 60 | 4.8(±0.2) | N |
| +O_2_ | 1 mM Zn | 120 | 6.2(±0.2) | N |

^a^ Δ*C*_q_ *_znuA_*_-_*_rpoD_* obtained for each biological replicate, mean (± standard deviation). Data from samples with a difference in *rpoD* *C*_q_ greater than 2 relative to untreated aerobic samples were not processed further, in samples treated with 1 mM ZnSO_4_ for 120 min this difference was not replicated with the use of an alternative control gene, *gyrA* (Supplementary Table 4, Methods).

**Supplementary Table 12**. Δ*C*_q_ values obtained from qPCR analysis of indicated samples with primers specific to *zntA* and *rpoD* (control)^a^.

| +/- O_2_ | Treatment | Exposure time (min) | Δ*C*_q_ *_zntA_*_-_*_rpoD_* | *rpoD* *C*_q_ difference relative to untreated aerobic samples <2? Yes/No |
| --- | --- | --- | --- | --- |
| +O_2_ | 2 mM Zn | 10 | -2.3(±0.1) | Y |
| +O_2_ | 0.6 mM Zn | 120 | -1.9(±0.2) | Y |
| +O_2_ | 1 mM Zn | 10 | -1.7(±0.3) | N |
| +O_2_ | 1 mM Zn | 60 | -0.82(±0.1) | N |
| +O_2_ | 1 mM Zn | 120 | -0.28(±0.3) | N |
| +O_2_ | Untreated | 120 | 1.2(±0.4) | n.a. |
| -O_2_ | Untreated | 120 | 1.7(±0.2) | Y |
| +O_2_ | 0.8 mM Zn | 120 | 1.8(±0.3) | Y |
| +O_2_ | 100 µM H_2_O_2_ | 120 | 2.7(±0.5) | Y |
| +O_2_ | 1 mM EDTA | 60 | 5.8(±0.2) | Y |
| +O_2_ | 1 mM EDTA | 120 | 6.5(±0.3) | Y |
| +O_2_ | 0.5 mM EDTA | 120 | 6.6(±0.2) | Y |
| +O_2_ | 1 mM EDTA | 20 | 7.0(±0.4) | N |
| +O_2_ | 1 mM EDTA | 240 | 7.3(±0.2) | Y |

^a^ Δ*C*_q_ *_zntA_*_-_*_rpoD_* obtained for each biological replicate, mean (± standard deviation). Data from samples with a difference in *rpoD* *C*_q_ greater than 2 relative to untreated aerobic samples were not processed further, in samples treated with 1 mM ZnSO_4_ for 120 min this difference was not replicated with the use of an alternative control gene, *gyrA* (Supplementary Table 4, Methods).

**Supplementary Table 13**. Δ*C*_q_ values obtained from qPCR analysis of indicated samples with primers specific to *copA* and *rpoD* (control)^a^.

| +/- O_2_ | Treatment | Exposure time (min) | Δ*C*_q_ *_copA_*_-_*_rpoD_* | *rpoD* *C*_q_ difference relative to untreated aerobic samples <2? Yes/No |
| --- | --- | --- | --- | --- |
| +O_2_ | 2.4 mM Cu | 10 | -5.8(±0.9) | N |
| +O_2_ | 2.4 mM Cu | 120 | -3.4(±0.07) | Y |
| +O_2_ | 1.8 mM Cu | 120 | -2.7(±0.3) | Y |
| -O_2_ | Untreated | 120 | -0.32(±0.2) | Y |
| +O_2_ | Untreated | 120 | 1.3(±0.2) | n.a. |
| +O_2_ | 1 mM EDTA | 60 | 1.4(±0.2) | Y |
| +O_2_ | 1 mM EDTA | 120 | 1.6(±0.1) | Y |
| +O_2_ | 0.5 mM EDTA | 120 | 1.8(±0.4) | Y |
| +O_2_ | 1 mM EDTA | 240 | 2.2(±0.2) | Y |
| +O_2_ | 100 µM H_2_O_2_ | 120 | 3.4(±0.3) | Y |
| +O_2_ | 1 mM EDTA | 20 | 3.8(±0.4) | N |

^a^ Δ*C*_q_ *_copA_*_-_*_rpoD_* obtained for each biological replicate, mean (± standard deviation). Data from samples with a difference in *rpoD* *C*_q_ greater than 2 relative to untreated aerobic samples were not processed further.

**Supplementary Table 14**. ΔΔ*C*_q_ values for conditional response of *mntS* promoter^a^.

| +/- O_2_ | Treatment | Exposure time (min) | ΔΔ*C*_q_ |
| --- | --- | --- | --- |
| +O_2_ | 1 mM EDTA | 60 | -6.7(±0.5) |
| +O_2_ | 1 mM EDTA | 240 | -6.3(±0.08) |
| +O_2_ | 1 mM EDTA | 120 | -6.1(±0.2) |
| +O_2_ | 0.5 mM EDTA | 120 | -5.9(±0.2) |
| +O_2_ | Untreated | 120 | -5.1(±0.7) |
| -O_2_ | Untreated | 120 | -4.6(±0.3) |
| +O_2_ | 0.5 mM Mn | 120 | -3.0(±0.3) |
| +O_2_ | 2 mM Mn | 120 | -2.8(±0.3) |
| +O_2_ | 4 mM Mn | 120 | -2.6(±0.4) |
| +O_2_ | 100 µM H_2_O_2_ | 60 | -1.9(±0.3) |
| +O_2_ | 100 µM H_2_O_2_ | 120 | -1.7(±0.3) |
| +O_2_ | 100 µM H_2_O_2_, 4 mM Mn | 120 | -0.48(±0.7) |
| +O_2_ | 100 µM H_2_O_2_, 4 mM Mn | 60 | - |

^a^ Determined by subtraction of Δ*C*_q_ for control condition (lowest expression, aerobic 60 min exposure to 100 µM H_2_O_2_ and 4 mM Mn, Supplementary Table 8) from Δ*C*_q_ for the condition of interest.

**Supplementary Table 15**. ΔΔ*C*_q_ values for conditional response of *fepD* promoter^a^.

| +/- O_2_ | Treatment | Exposure time (min) | ΔΔ*C*_q_ |
| --- | --- | --- | --- |
| +O_2_ | 1 mM EDTA | 60 | -6.7(±0.1) |
| +O_2_ | 1 mM EDTA | 120 | -6.2(±0.6) |
| +O_2_ | 0.5 mM EDTA | 120 | -5.9(±0.3) |
| +O_2_ | 1 mM EDTA | 240 | -5.7(±0.1) |
| -O_2_ | Untreated | 120 | -2.5(±0.4) |
| -O_2_ | 0.5 mM Fe | 120 | -1.6(±0.04) |
| +O_2_ | Untreated | 120 | -0.91(±0.2) |
| +O_2_ | 0.5 mM Fe | 120 | -0.63(±0.1) |
| +O_2_ | 100 µM H_2_O_2_ | 120 | - |

^a^ Determined by subtraction of Δ*C*_q_ for control condition (lowest expression, aerobic 120 min exposure to 100 µM H_2_O_2_, Supplementary Table 9) from Δ*C*_q_ for the condition of interest.

**Supplementary Table 16**. ΔΔ*C*_q_ values for conditional response of *rcnA* promoter^a^.

| +/- O_2_ | Treatment | Exposure time (min) | ΔΔ*C*_q_ |
| --- | --- | --- | --- |
| +O_2_ | 0.5 mM Co | 120 | -6.6(±0.4) |
| +O_2_ | 1 mM EDTA | 60 | -3.3(±0.2) |
| +O_2_ | 1 mM EDTA | 120 | -3.0(±0.2) |
| +O_2_ | 1 mM EDTA | 240 | -2.7(±0.3) |
| +O_2_ | 0.5 mM EDTA | 120 | -2.4(±0.2) |
| -O_2_ | Untreated | 120 | -1.6(±0.09) |
| +O_2_ | Untreated | 120 | -1.4(±0.3) |
| +O_2_ | 100 µM H_2_O_2_ | 120 | - |

^a^ Determined by subtraction of Δ*C*_q_ for control condition (lowest expression, aerobic 120 min exposure to 100 µM H_2_O_2_, Supplementary Table 10) from Δ*C*_q_ for the condition of interest.

**Supplementary Table 17**. ΔΔ*C*_q_ values for conditional response of *nikA* promoter under aerobic conditions^a^.

| +/- O_2_ | Treatment | Exposure time (min) | ΔΔ*C*_q_ |
| --- | --- | --- | --- |
| +O_2_ | 1 mM EDTA | 60 | -5.6(±0.5) |
| +O_2_ | 1 mM EDTA | 120 | -5.1(±0.4) |
| +O_2_ | Untreated | 120 | -4.9(±0.4) |
| +O_2_ | 1 mM EDTA | 240 | -4.4(±0.5) |
| +O_2_ | 0.5 mM EDTA | 120 | -3.8(±0.5) |
| +O_2_ | 100 µM H_2_O_2_ | 120 | -3.6(±0.3) |
| +O_2_ | 0.5 mM Ni | 120 | - |

^a^ Determined by subtraction of Δ*C*_q_ for control condition (lowest expression, aerobic 120 min exposure to 0.5 mM Ni, Supplementary Table 7) from Δ*C*_q_ for the condition of interest.

**Supplementary Table 18**. ΔΔ*C*_q_ values for conditional response of *nikA* promoter under anaerobic conditions^a^.

| +/- O_2_ | Treatment | Exposure time (min) | ΔΔ*C*_q_ |
| --- | --- | --- | --- |
| -O_2_ | 1 mM DMG | 120 | -5.7(±0.2) |
| -O_2_ | 0.5 mM DMG | 120 | -5.6(±0.3) |
| -O_2_ | 1 mM EDTA | 120 | -5.5(±0.2) |
| -O_2_ | Untreated | 120 | -5.2(±0.1) |
| -O_2_ | 1.5 mM Ni | 120 | -1.5(±0.1) |
| -O_2_ | 0.5 mM Ni | 120 | -0.84(±0.4) |
| -O_2_ | 2 mM Ni | 120 | - |

^a^ Determined by subtraction of Δ*C*_q_ for control condition (lowest expression, anaerobic 120 min exposure to 2 mM Ni, Supplementary Table 7) from Δ*C*_q_ for the condition of interest.

**Supplementary Table 19**. ΔΔ*C*_q_ values for conditional response of *znuA* promoter^a^.

| +/- O_2_ | Treatment | Exposure time (min) | ΔΔ*C*_q_ |
| --- | --- | --- | --- |
| +O_2_ | 1 mM EDTA | 60 | -6.4(±0.7) |
| +O_2_ | 1 mM EDTA | 240 | -6.3(±0.1) |
| +O_2_ | 1 mM EDTA | 120 | -6.2(±0.3) |
| +O_2_ | 0.8 mM Zn | 120 | -6.0(±0.4) |
| +O_2_ | 0.5 mM EDTA | 120 | -5.7(±0.8) |
| +O_2_ | 0.6 mM Zn | 120 | -2.6(±0.1) |
| -O_2_ | Untreated | 120 | -2.2(±0.2) |
| +O_2_ | Untreated | 120 | -2.1(±0.3) |
| +O_2_ | 100 µM H_2_O_2_ | 120 | - |

^a^ Determined by subtraction of Δ*C*_q_ for control condition (lowest expression, aerobic 120 min exposure to 100 µM H_2_O_2_, Supplementary Table 11) from Δ*C*_q_ for the condition of interest.

**Supplementary Table 20**. ΔΔ*C*_q_ values for conditional response of *zntA* promoter^a^.

| +/- O_2_ | Treatment | Exposure time (min) | ΔΔ*C*_q_ |
| --- | --- | --- | --- |
| +O_2_ | 2 mM Zn | 10 | -9.6(±0.1) |
| +O_2_ | 0.6 mM Zn | 120 | -9.1(±0.2) |
| +O_2_ | Untreated | 120 | -6.1(±0.4) |
| -O_2_ | Untreated | 120 | -5.6(±0.2) |
| +O_2_ | 0.8 mM Zn | 120 | -5.5(±0.3) |
| +O_2_ | 100 µM H_2_O_2_ | 120 | -4.5(±0.5) |
| +O_2_ | 1 mM EDTA | 60 | -1.4(±0.2) |
| +O_2_ | 1 mM EDTA | 120 | -0.7(±0.3) |
| +O_2_ | 0.5 mM EDTA | 120 | -0.7(±0.2) |
| +O_2_ | 1 mM EDTA | 240 | - |

^a^ Determined by subtraction of Δ*C*_q_ for control condition (lowest expression, aerobic 240 min exposure to 1 mM EDTA, Supplementary Table 12) from Δ*C*_q_ for the condition of interest.

**Supplementary Table 21**. ΔΔ*C*_q_ values for conditional response of *copA* promoter^a^.

| +/- O_2_ | Treatment | Exposure time (min) | ΔΔ*C*_q_ |
| --- | --- | --- | --- |
| +O_2_ | 2.4 mM Cu | 120 | -6.8(±0.07) |
| +O_2_ | 1.8 mM Cu | 120 | -6.0(±0.3) |
| -O_2_ | Untreated | 120 | -3.7(±0.2) |
| +O_2_ | Untreated | 120 | -2.1(±0.2) |
| +O_2_ | 1 mM EDTA | 60 | -2.0(±0.2) |
| +O_2_ | 1 mM EDTA | 120 | -1.8(±0.1) |
| +O_2_ | 0.5 mM EDTA | 120 | -1.6(±0.4) |
| +O_2_ | 1 mM EDTA | 240 | -1.2(±0.2) |
| +O_2_ | 100 µM H_2_O_2_ | 120 | - |

^a^ Determined by subtraction of Δ*C*_q_ for control condition (lowest expression, aerobic 120 min exposure to 100 µM H_2_O_2_, Supplementary Table 13) from Δ*C*_q_ for the condition of interest.

**Supplementary Table 22**. Percentage similarity and identity between metal sensors of *E. coli* JM109 (DE3) and *Salmonella*^a^.

| Protein | Similarity (%) | Identity (%) |
| --- | --- | --- |
| MntR | 93.0 | 89.8 |
| Fur | 97.3 | 96.7 |
| RcnR | 96.7 | 93.3 |
| NikR | 99.2 | 98.5 |
| Zur | 95.3 | 93.0 |
| ZntR | 97.9 | 92.2 |
| CueR | 94.2 | 89.1 |

^a^ We do not note any variation for proposed metal binding residues between the *E. coli* and *Salmonella* proteins. Additionally, we note that residues required for allostery and DNA binding are conserved in MntR and Zur. Pairwise comparison performed with EMBOSS Needle (<https://www.ebi.ac.uk/Tools/psa/emboss_needle/>) [17].


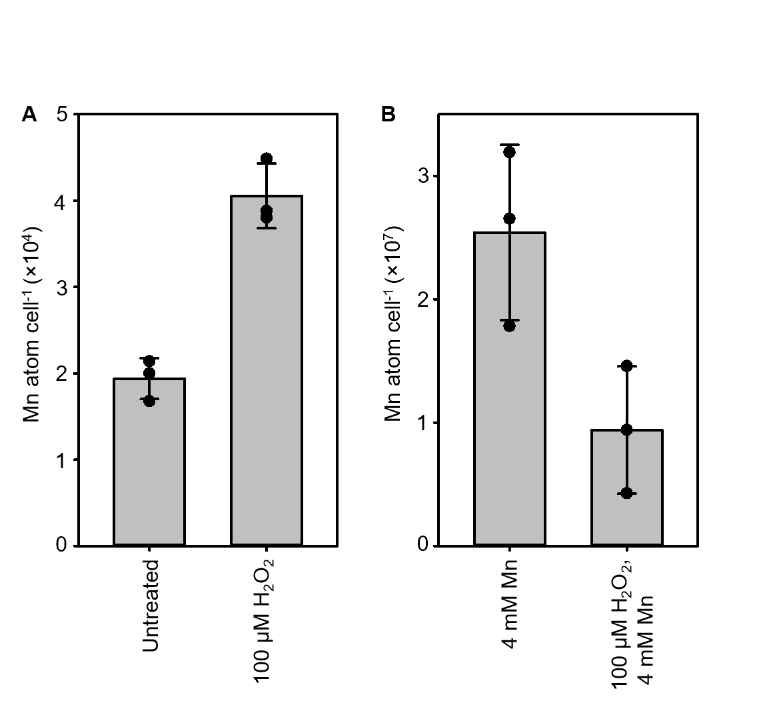


**Supplementary Figure 1.** **Total cellular manganese content of *E. coli* JM109(DE3).** Cells were cultured aerobically with a 2 h exposure to the indicated treatment before harvest (n = 3 biological replicates per treatment). Cell pellets were washed with 10 mM HEPES pH 7.8, 0.5 M sorbital, 1 mM EDTA followed by the same buffer without EDTA and digested in 65% HNO_3_ before analysis by ICP-MS. OD_600 nm_ values at harvest were converted to cells per ml using the approximation OD_600 nm_ = 1 correlates to 4.4 × 10^8^ cell ml^-1^ [2]. Data is presented as mean ± standard deviation. Note the magnitude of scale difference between **A** and **B**.

**References**

1 Kaluarachchi, H., Siebel, J. F., Kaluarachchi-Duffy, S., Krecisz, S., Sutherland, D. E., Stillman, M. J. & Zamble, D. B. Metal selectivity of the *Escherichia coli* nickel metallochaperone, SlyD. *Biochemistry* **50**, 10666-10677 (2011).

2 Young, T. R., Martini, M. A., Foster, A. W., Glasfeld, A., Osman, D., Morton, R. J., Deery, E., Warren, M. J. & Robinson, N. J. Calculating metalation in cells reveals CobW acquires Co(II) for vitamin B12 biosynthesis while related proteins prefer Zn(II). *Nat. Commun.* **12**, 1195 (2021).

3 Kehres, D. G., Zaharik, M. L., Finlay, B. B. & Maguire, M. E. The NRAMP proteins of Salmonella typhimurium and Escherichia coli are selective manganese transporters involved in the response to reactive oxygen. *Mol. Microbiol.* **36**, 1085-1100 (2000).

4 Kehres, D. G., Janakiraman, A., Slauch, J. M. & Maguire, M. E. Regulation of *Salmonella enterica* serovar Typhimurium mntH transcription by H2O2, Fe2+, and Mn2+. *J. Bacteriol.* **184**, 3151-3158 (2002).

5 Beauchene, N. A., Myers, K. S., Chung, D., Park, D. M., Weisnicht, A. M., Keleş, S. & Kiley, P. J. Impact of anaerobiosis on expression of the iron-responsive Fur and RyhB regulons. *MBio* **6**, e01947-01915 (2015).

6 Beauchene, N. A., Mettert, E. L., Moore, L. J., Keleş, S., Willey, E. R. & Kiley, P. J. O2 availability impacts iron homeostasis in Escherichia coli. *Proc. Natl. Acad. Sci.* **114**, 12261-12266 (2017).

7 Benoit, S. L., Schmalstig, A. A., Glushka, J., Maier, S. E., Edison, A. S. & Maier, R. J. Nickel chelation therapy as an approach to combat multi-drug resistant enteric pathogens. *Scientific reports* **9**, 1-10 (2019).

8 Waters, L. S., Sandoval, M. & Storz, G. The *Escherichia coli* MntR miniregulon includes genes encoding a small protein and an efflux pump required for manganese homeostasis. *J. Bacteriol.* **193**, 5887-5897 (2011).

9 Iwig, J. S., Rowe, J. L. & Chivers, P. T. Nickel homeostasis in *Escherichia coli* – the rcnR‐rcnA efflux pathway and its linkage to NikR function. *Mol. Microbiol.* **62**, 252-262 (2006).

10 De Pina, K., Desjardin, V., Mandrand-Berthelot, M. A., Giordano, G. & Wu, L. F. Isolation and Characterization of the *nikR* Gene Encoding a Nickel-Responsive Regulator in *Escherichia coli*. *J. Bacteriol.* **181**, 670-674 (1999).

11 Chivers, P. T. & Sauer, R. T. Regulation of high affinity nickel uptake in bacteria: Ni(II)-dependent interaction of NikR with wild-type and mutant operator sites. *J. Biol. Chem.* **275**, 19735-19741 (2000).

12 Outten, F. W., Outten, C. E., Hale, J. & O'Halloran, T. V. Transcriptional Activation of an *Escherichia coli* Copper Efflux Regulon by the Chromosomal MerR Homologue, CueR. *J. Biol. Chem.* **275**, 31024-31029 (2000).

13 Outten, C. E., Outten, F. W. & O'Halloran, T. V. DNA distortion mechanism for transcriptional activation by ZntR, a Zn (II)-responsive MerR homologue in *Escherichia coli*. *J. Biol. Chem.* **274**, 37517-37524 (1999).

14 Gilston, B. A., Wang, S., Marcus, M. D., Canalizo-Hernández, M. A., Swindell, E. P., Xue, Y., Mondragón, A. & O'Halloran, T. V. Structural and mechanistic basis of zinc regulation across the *E. coli* Zur regulon. *PLoS biology* **12**, e1001987 (2014).

15 Seo, S. W., Kim, D., Latif, H., O’Brien, E. J., Szubin, R. & Palsson, B. O. Deciphering Fur transcriptional regulatory network highlights its complex role beyond iron metabolism in *Escherichia coli*. *Nat. Commun.* **5**, 1-10 (2014).

16 Osman, D., Martini, M. A., Foster, A. W., Chen, J., Scott, A. J. P., Morton, R. J., Steed, J. W., Lurie-Luke, E., Huggins, T. G., Lawrence, A. D., Deery, E., Warren, M. J., Chivers, P. T. & Robinson, N. J. Bacterial sensors define intracellular free energies for correct enzyme metalation. *Nat. Chem. Biol.* **15**, 241-249 (2019).

17 Needleman, S. B. & Wunsch, C. D. A general method applicable to the search for similarities in the amino acid sequence of two proteins. *J. Mol. Biol.* **48**, 443-453 (1970).
